# Strengthening coordination and collaboration of one health approach for zoonotic diseases in Africa

**DOI:** 10.1186/s42522-023-00082-5

**Published:** 2023-08-02

**Authors:** Yewande Alimi, James Wabacha

**Affiliations:** 1 Africa Centres for Disease Control and Prevention, Addis Ababa, Ethiopia; 2 African Union InterAfrican Bureau for Animal Resources, Nairobi, Kenya

**Keywords:** One health, Africa, African Union, Zoonoses

## Abstract

Despite the One Health progress made in some African countries in addressing zoonotic disease outbreaks, many still lack formal and funded One Health programs. Countries lack diagnostic capacity for zoonotic diseases, coordinated surveillance mechanisms, multisectoral response strategies and skilled workforce. With the devasting impacts of zoonotic disease outbreaks, recent epidemics have caused a loss of lives and negatively impacted the economy. Strengthening One Health approach across African Union (AU) Member States will improve the continent’s ability and capacity to efficiently prevent, detect, and respond to emerging and re-emerging zoonotic diseases. The policy and practice changes needed to address zoonotic diseases require strong political commitment, financial investments, and institutionalised national One Health programs. The African Union endorses a One Health approach in which multiple sectors work jointly to raise awareness, gather credible data, implement programs, and promote evidence-based policy and practice in improve human, animal, and environmental health. The African Union working through its technical agencies set up an interagency multidisciplinary group “the One Health Coordinating Group on Zoonotic Diseases” to strengthen coordinated surveillance, prevention and control of zoonotic diseases on the continent. There is an urgent need to strengthen the coordination of One Health activities across the African continent. The African Union will leverage its unique political position on the continent to raise awareness, secure commitments, and influence policy at the head of state level. This manuscript highlights the opportunity to improve and strengthen One Health coordination and harmonisation of efforts through a continental strategy for zoonotic disease control.

## Background

Novel pathogens continue to emerge globally, the majority of which have an animal origin and zoonotic potential, meaning that they are transmissible between animals and humans [1]. About 60% of existing and 75% of newly emerging infectious diseases in humans are zoonotic and majority of these diseases originate from wildlife [2, 3].

Emerging zoonotic diseases are gravely concerning and pose both a significant and growing threat to global health, global security and the economy [4, 5]. Six of the seven Public Health Emergencies of International Concern (PHEICs) are zoonotic – the Influenza A virus subtype H1N1 (H1N1) 2009 H1N1 pandemic, the 2014-16 Ebola outbreaks in West Africa, the 2015–16 Zika virus outbreaks, the 2018–20 Kivu Ebola epidemic, the COVID-19 pandemic, and the 2022 monkeypox outbreak. These events and other zoonotic outbreaks have resulted in billions of dollars lost and indirect losses affecting economies [6]. The economic impacts go beyond the public health sector to include the tourism, agricultural, and financial sectors [5]. In many low- and middle-income countries, it is estimated that annually zoonotic diseases result in 2.5 billion cases of human illness and 2.7 million deaths [7].

Human actions have severely altered 75% of terrestrial environments and 66% of marine environments, with a 300% increase in crop agricultural crop production since 1970 [8]. As humans and livestock continue to encroach into natural habitats of wildlife, either by urbanization, tourism or wildlife hunting, agriculture intensification practices reducing biodiversity, there is an increased frequency and intimacy between humans, domestic animals and wild animals, contributing to the emergence of zoonotic diseases [9, 10].

The probability of emergence of zoonotic pathogens from wildlife to humans is positively correlated to wildlife biodiversity and growing human population [3, 10]. Further, there is evidence to support that disease emergence is largely driven by changes such as the mentioned increased urbanisation, expanded agriculture practices/ intensification along with globalisation, wildlife hunting and encroachment, deforestation, as well as climate change [11–13]. As climate change progresses, the number of emerging zoonotic events is estimated to continue to increase, disproportionately impacting Asia and Africa [14].

Human activities such as wildlife hunting and bushmeat trade for human consumption, which often serves as a source of protein for rural communities in low-and-middle income countries [15], leads to increased human-animal interaction in live animal markets, wildlife/petting farms and within the wildlife trade increasing the risk for novel pathogen emergence from wildlife [16, 17], while great ape tourism increases the risk of reverse zoonotic disease transmission [18].

New agricultural practices such as mink farming highlights the role of humans in reverse zoonosis: the mink farm SARS-COV-2 outbreaks were a result of a “spill over” from the humans who worked on the mink farms. In Africa and Asia, intensive farming practices linked to urban landscapes management creates a unique opportunity for outbreaks of emerging and re-emerging pathogens [19].

## Africa’s experiences with dealing with zoonoses

Many globally important zoonoses such as rabies, anthrax, brucellosis, cysticercosis are endemic especially amongst the poorest communities in Africa [20]. The African continent disproportionally experiences more outbreaks than the rest of the world, especially zoonotic outbreaks ranging from endemic zoonoses such as brucellosis and leptospirosis to neglected zoonoses such as rabies and onchocerciasis to emerging zoonoses such as anthrax, yellow fever, Ebola, Lassa fever and COVID-19 [21]. A meta-analysis reports a 61% case fatality rate of bat-originated viral zoonotic diseases in Africa [22]. Specifically, rabies results in an estimated 21,000 deaths per year across the African continent and major losses in African countries with livestock-dependent economies [23, 24].

The continent has both competent vectors, high biodiversity and environmental conditions that support propagation of zoonotic diseases with pandemic potential like zoonotic influenza viruses and viral haemorrhagic fevers like Ebola, Marburg, Lassa fever, and Rift Valley fever. Across the continent, outbreaks of anthrax, Ebola, zoonotic influenza, monkeypox, Lassa Fever, and Rift Valley fever continue to cause severe illness and death in humans and animals, impact livelihoods, disrupt movement of goods and people, lead to food insecurity, strain national health systems, and result in massive economic losses for both the government and private sector.

## Breaking silos for a safer Africa

Given the interconnectedness of the health of humans, animals and environment the multiplicity of players needs to address the risks at the interface, within the context of scarce resources in Africa. Zoonotic disease prevention actions cost less than 1/20th the value of lives lost each year and therefore presents a business case to address the heavy burden of both emerging, re-emerging and endemic zoonoses in the continent and globally [4].

The continent’s governing body; the African Union (AU) is comprised of the 55 Member States that make up the countries of the continent, with the main objectives of promoting unity and solidarity amongst its Member States; to coordinate and intensify cooperation for development; and to promote international cooperation. The Pan-African vision of an integrated, prosperous and peaceful Africa is anchored to the Agenda 2063.

A One Health approach will improve our ability to efficiently prevent, detect, and respond to emerging and re-emerging zoonotic diseases at the human–animal–environment interface [25]. Practically, One Health involves the collaboration between human, animal, and environmental health sectors as well as other relevant stakeholders, in the design and implementation of programmes, policies, legislation, and research intended to achieve better health outcomes for all [26].

Operationalising a One Health approach involves the effective collaboration between human, animal and environmental health and all relevant sectors to address shared health threats. African Union Member States have documented remarkable progress in institutionalising One Health mechanisms for zoonotic disease control and prevention.

Despite the known benefits and progress that has been made over the past decade, institutionalization and operationalization of One Health can be challenging, including breaking down established professional and programmatic silos that currently exist within government and non-governmental agencies and institutions.

There is a significant gap in political commitment, skilled workforce, domestic financing and legal frameworks to strengthen coordination, collaboration, and communication among One Health stakeholders in Africa [27]. Leveraging the lessons learned from embracing a regional approach in fighting the COVID-19 pandemic in Africa, it is the imperative that the African Union through its technical and regional bodies implement and support One Health institutionalisation across the African Union Member states to effectively prevent and respond to zoonotic disease outbreaks to achieve Agenda 2063: The Africa We Want”.

Recognising the urgent need for action, in 2010, the World Health Organization (WHO), Food and Agriculture Organization of the United Nations (FAO), and World Organisation for Animal Health (WOAH); the Tripartite, formalized a multidisciplinary collaboration to address health threats at the human–animal–ecosystem interface [28]. To ensure a holistic One Health approach, the Tripartite organisations and the UN Environment Programme (UNEP) signed a ground breaking Memorandum of Understanding forming a new Quadripartite Collaboration for One Health in 2022. (Quadripartite Memorandum of Understanding (MoU) signed for a new era of One Health collaboration, 2022) The Quadripartite has also adopted a One Health definition to aid interpretation harmonisation, understanding and implementation of One Health [29].

## A Continental push for one health

In Africa, several AU Member States have embraced a One Health approach to prevent and control shared health threats like zoonotic diseases [30], this approach should be widely adopted in practice and institutionalised through national frameworks and One Health programs [32].

The AU recognizes that preventing and controlling the outbreaks of zoonotic disease will require scientific knowledge and changes in policy and practice across multiple sectors. The AU endorses the One Health approach to address zoonotic diseases, human, animal, and environmental sectors work jointly to raise awareness, gather credible data, implement programs, and promote evidence-based policy and practice. African Union organs have begun implementing programs to address zoonotic diseases including the Africa Centres for Disease Control and Prevention (Africa CDC), Inter-African Bureau for Animal Resources (AU-IBAR), African Union Pan-African Veterinary Vaccine Centre (AU-PANVAC), The New Partnership for Africa’s Development (NEPAD), The Citizens and Diaspora Organizations (CIDO), Scientific, Technical and Research Commission (STRC), Inter-African Phytosanitary Council (IAPSC), Pan African Tsetse and Trypanosomiasis Eradication Campaign (PATTEC), Education, Science, Technology and Innovation Department; Health, Humanitarian Affairs and Social Development (Division of Health Systems, Diseases and Nutrition); Directorate for Sustainable Environment and Blue Economy (SEBE); Women, Gender and Youth Directorate; and AU Regional Economic Communities.

The Africa Centres for Disease Control and Prevention (Africa CDC) in support of strengthening the One Health approach across the continent, has led the development and is supporting Member States with the implementation of a Framework for One Health Practice in National Public Health Institutes (NPHIs) that focuses on zoonotic disease prevention and control. The Framework aims to improve One Health multisectoral coordination; strengthen coordinated surveillance systems and data sharing mechanisms; strengthen diagnostic capacity and laboratory networks; improve multisectoral public health preparedness and response; and scale up multisectoral workforce [31], aligned to the Africa CDC’s mission to strengthen Africa’s public health institutions’ capacities, capabilities and partnerships to detect and respond quickly and effectively to disease threats and outbreaks based on science, policy, and data-driven interventions and programs. While other One Health relevant health challenges such as around antimicrobial resistance (AMR) and Food Safety are being addressed, as well, priority is currently placed on zoonotic diseases due to the described significant impacts on the African continent.

‌Over the past decade, AU-IBAR has developed and implemented programs and projects with a One Health approach and spearheaded efforts for its institutionalization by both AU member states and regional economic communities RECs [32]. Given its continental mandate for animal resources, AU-IBAR has supported the development and implementation of the integrated National Actions Plans against highly pathogenic avian influenza (HPAI) and multi-sectoral interventions. AU-IBAR continues to support coordination and harmonisation of One Health interventions in the continent.

Institutionalising One Health requires strong governance political leadership, and community engagement to sustain the multi-sectoral and interdisciplinary collaborations, and formalise One Health coordinating mechanisms to improve data sharing and decision-making. Recognising the need to improve and strengthen One Health activities, coordination and harmonisation of efforts across AU member states and with partners supporting One Health activities, there is a need to develop an AU One Health Strategy for Zoonotic Disease Prevention and Control.

The continental strategy will leverage on the AU’s convening power and advocate for political support for One Health at national, regional and continental levels to improve countries’ ability to prevent, detect, and respond to emerging zoonotic disease threats. The newly established AU One Health Coordination Group on Zoonotic Diseases will lead and facilitate the strategy development process together with Member States and will work on further related objectives to support One Health approaches on the continent.

## Conclusions

Humankind has faced several “apocalyptic extinctions” during the Black Death of the fourteenth century in Europe, the 1918–1919 influenza A pandemic and, most recently the COVID-19 pandemic. Emerging zoonotic diseases are gravely concerning and pose a significant growing threat to global health, global security and economy.

The One Health approach is a solution to prevent and counter the emergence of zoonotic diseases and other shared public health threats. A One Health approach is critical for the accelerated implementation of the International Health Regulations (IHR 2005), the WOAH International Standards to safeguard the socioeconomic and political integration of the continent and to achieve the AU Agenda 2063: The Africa We Want. The African Union endorses the One Health approach to address zoonotic diseases, human, animal, and environmental sectors work jointly to raise awareness, gather credible data, implement programs, and promote evidence-based policy and practice.

Operationalising One Health remains a challenge and has struggled to gain a firm institutional foothold as One Health activities continue to exist in silos. To help eliminate the preceding siloed approach of addressing zoonotic diseases at national level, the AU organisations are leading the development of an African Union One Health strategy to support AU Member States to develop/establish, coordinate, monitor, and evaluate implementation of holistic zoonotic disease prevention and control programmes and help get ahead and prevent future pandemics.

The African Union envisions and strives for an integrated and prosperous Africa free of disease, disability, and premature death and aims to promote the well-being of all in Africa through “Agenda 2063: The Africa We Want” using the One Health approach.

## Data Availability

not applicable.
